# The incidence of hyperkalemia in patients with secondary hyperparathyroidism after ultrasound-guided radiofrequency ablation vs. parathyroidectomy

**DOI:** 10.3389/fmed.2025.1539652

**Published:** 2025-02-18

**Authors:** Mian Ren, Yueming Liu, Bo Lin, Wenli Zou, Bin Zhu, Juan Wu

**Affiliations:** Urology & Nephrology Center, Department of Nephrology, Zhejiang Provincial People's Hospital (Affiliated People's Hospital), Hangzhou Medical College, Hangzhou, Zhejiang, China

**Keywords:** secondary hyperparathyroidism, maintenance dialysis, parathyroidectomy, ultrasound-guided radiofrequency ablation, hyperkalemia

## Abstract

**Introduction:**

The aim of our study was to compare the incidence of hyperkalemia in maintenance dialysis patients with secondary hyperparathyroidism(SHPT) after parathyroidectomy(PTX) or ultrasound-guided radiofrequency ablation(RFA), and to explore the risk factors and the best preoperative serum potassium threshold.

**Methods:**

We defined hyperkalemia as serum potassium >5.30 mmol/L. Two operation methods were analyzed in subgroups, and the differences in demographic parameters, operation situation and perioperative laboratory indicators were compared between the two groups.

**Results:**

A total of 154 maintenance dialysis patients were included, of which 91 patients received PTX and 63 patients received RFA. 53 patients (34.4%) developed postoperative hyperkalemia. Patients in hyperkalemia group had higher preoperative serum potassium, phosphorus, hemoglobin and 25-hydroxyvitamin D level (*p* < 0.05). It seemed that males were more prone to suffer hyperkalemia than females, but there was no statistically difference (40.9% vs. 25.8%, *p* = 0.05). The occurrence of hyperkalemia after the operation was obviously higher in hemodialysis patients. Logistic regression analysis showed that preoperative serum potassium level (OR = 3.269, 95%CI 1.638–6.534, *p* = 0.001) and PTX (OR = 18.119, 95%CI 5.716–57.438, *p* < 0.01) were independent risk factors for predicting postoperative hyperkalemia. According to ROC curve analysis, the optimal cutoff value for preoperative serum potassium was 4.66 mmol/L, with a sensitivity of 46.8% and a specificity of 86%.

**Conclusion:**

Hyperkalemia after invasive treatment in patients with severe SHPT was common, and the incidence of hyperkalemia after PTX was significantly higher than that after RFA. Hemodialysis patients were more prone to hyperkalemia, which was related to the preoperative serum potassium level.

## Introduction

Persistent calcium-phosphorus-vitamin D and parathyroid axis metabolism disorders due to progressive renal failure, which ultimately triggers secondary hyperparathyroidism (SHPT) (1–3), is one of the common complications in patients with end-stage renal disease (ESRD). Clinical manifestations included abnormal bone mineral metabolism, renal osteodystrophy, and vascular calcification, with a consequent increased risk of cardiovascular events and death.

For patients with severe drug-invalid SHPT, ultrasound-guided radiofrequency ablation (RFA) (4, 5)and total parathyroidectomy (PTX) (6–8)are both effective and invasive treatment. The incidence of intraoperative and postoperative hyperkalemia after PTX has been reported to be 25–80% (9–13), which may lead to numbness of limbs, fatigue, vomiting, dyspnea, arrhythmias and even lethal consequences. However, the influencing factors and mechanisms of perioperative hyperkalemia are not clear (14). Therefore, the aim of our retrospective study was to investigate and compare the incidence of hyperkalemia after the therapy of PTX or RFA in ESRD patients with SHPT in our center, and to explore the risk factors for hyperkalemia and the optimal preoperative serum potassium cutoff value in order to avoid adverse events.

## Materials and methods

### Patients

This study was approved by the Bioethics Committee of Zhejiang Provincial People’s Hospital (Code: QT2024036). Ethical approval exempts informed consent. We confirm that all methods were performed in accordance with the relevant guidelines and regulations of declarations of Helsinki. The retrospective cohort study included all patients diagnosed as SHPT who underwent PTX or ultrasound-guided RFA from June 2014 to December 2022 in our hospital. The inclusion criteria were (1) 18–85 years old; (2) dialytic vintage ≥6 months; (3) postoperative iPTH concentration > 600 pg./mL; (4) severe SHPT after ineffective medical treatment; (5) at least one hyperplastic parathyroid nodules with diameter ≥ 1 cm found by ultrasound examination; (6) follow-up durations ≥3 months; The exclusion criteria were: (1) primary or tertiary hyperparathyroidism; (2) severe cardiopulmonary insufficiency who cannot tolerate treatment; (3) previous history of PTX or RFA;

### Intervention

The operation processes of both invasive treatments were described in our previous study (15).

Drugs for induction of general anesthesia in total PTX: propofol 0.5 g, remifentanil 1 mg, etomidate 10 mg, remazolam benzene sulfonate 5 mg, atracurium benzenesulfonate 12 mg, pentazocine 15 mg, dexamethasone 5 mg, pentylenetetrazol 0.3 mg, palonosetron 0.25 mg. And RFA was performed after local anesthesia by 0.1 g lidocaine.

All enrolled hemodialysis patients received heparin-free dialysis on the day before treatment and on 1 week postoperatively. Peritoneal dialysis patients switched to hemodialysis temporarily if needed, otherwise they continued the original peritoneal dialysis. We defined hyperkalemia as serum potassium >5.30 mmol/L and hypocalcemia as serum calcium <2.0 mmol/L. And emergency hemodialysis was initiated in case of hyperkalemia monitored. The dialysate calcium concentration was 1.5 mmol/ L.

### Clinical data collection

General information of age, gender, dialysis history, clinical symptoms and treatment (cinacalcet therapy) were collected. Clinical laboratory variables at baseline such as serum creatinine (Cr), uric acid (UA), albumin (ALB), hemoglobin (Hb), troponin I (TNI), B-type natriuretic peptide (BNP), C-reactive protein (CRP), intact parathyroid hormone (iPTH), calcium (Ca), phosphorus (P), potassium (K) and magnesium (Mg) levels, and bone metabolism related indicators such as alkaline phosphatase (ALP), Beta C-terminal cross-linked telopeptides of type I collagen (*β*-CTx), N-terminal osteocalcin (N-MID), total type I collagen N-terminal propeptide (tP1NP), 25-hydroxy vitamin D (25(OH)D), etc. were collected. Clinical data including the number and size of parathyroid nodules, osteoporosis or not (bone density), carotid arteriosclerosis or not (carotid artery B-ultrasound), hospital stay and postoperative complications. The duration of operation (the time between incision and suture) and the number of parathyroid nodules removed were recorded.

### Follow-up and outcomes

Postoperative serum potassium and pH levels were conducted. The primary outcome was the occurrence of perioperative hyperkalemia in our center. The patients were divided into hyperkalemia group and non-hyperkalemia group. We investigated the potential risk factors led to perioperative hyperkalemia and explored the optimal preoperative potassium cutoff value in order to reduce the incidence of hyperkalemia. The secondary outcome was the differences in the changes of iPTH, calcium and phosphorus concentrations over time between PTX or RFA group.

### Statistical analysis

All statistical analyses were performed using SPSS version 26.0 for Mac and Graphpad Prism version 9.0. The measured data conforming to a normal distribution were displayed as the mean ± standard deviation (SD), and the other data were displayed as the median and interquartile range. Comparisons between parameters were performed using the independent sample T-test, Mann–Whitney U test or Chi-squared test. Potential predictors of hyperkalemia occurrence were analyzed by logistical regression analysis. According to receiver operating characteristic (ROC) curve analysis, the best cutoff point of preoperative serum potassium was calculated. All results were tested by bilateral tests, and significance was indicated by *p* < 0.05.

## Results

### General information

A total of 154 patients were treated from June 2014 to December 2022. There were 91 patients in the PTX group, of whom 85 (93.4%) underwent total parathyroidectomy with autotransplantation; 6 (6.6%) underwent total parathyroidectomy only, and 63 were in the RFA group. The baseline and clinical data of the patients are summarized in Table 1. The average age of these patients was 52.10 ± 13.56 years old, the average dialytic vintage was 7.75 ± 3.61 years, and 57.1% were males. A total of 125 (81.2%) patients were undergoing maintenance hemodialysis, and the others were undergoing peritoneal dialysis.

**Table 1:** Patients’ baseline characteristics.

Parameter	Hyperkalemia Group (*n* = 53)	Non-hyperkalemia Group (*n* = 101)	*p* value
Pre-operative data			
Age(years)	52.15 ± 12.56	52.08 ± 14.12	0.98
Female:male(n/n)	17/36	49/52	0.05
Dialysis vintage(years)	8.14 ± 3.71	7.55 ± 3.56	0.33
Dialysis method, hemodialysis	90.56%	76.23%	0.03
Albumin(g/L)	36.61 ± 3.95	35.97 ± 4.90	0.42
Creatinine(μmol/L)	881.09 ± 252.20	833.04 ± 221.13	0.22
Uric acid (μmol/L)	415.51 ± 95.51	414.17 ± 110.73	0.94
TnI(μg/L)	0.03 ± 0.04	0.04 ± 0.05	0.18
BNP(pg/ml)	522.71 ± 909.94	554.12 ± 825.39	0.84
Hemoglobin (g/L)	110.47 ± 23.55	102.72 ± 18.94	0.03
ALP(U/L)	376.38 ± 317.51	386.19 ± 365.63	0.87
β-CTx(pg/ml)	5324.96 ± 1087.81	5452.85 ± 953.72	0.46
N-MID(ng/ml)	279.12 ± 388.89	231.90 ± 61.17	0.26
tPINP(ng/ml)	1068.86 ± 337.89	1089.27 ± 232.25	0.70
25(OH)D(ng/ml)	25.47 ± 11.20	21.28 ± 11.96	0.04
Magnesium(mmol/L)	1.06 ± 0.23	1.05 ± 0.25	0.75
pH	7.40 ± 0.05	7.41 ± 0.05	0.41
Potassium (mmol/L)	4.54 ± 0.74	4.21 ± 0.59	<0.01
Calcium (mmol/L)	2.42 ± 0.19	2.44 ± 0.26	0.65
iPTH (pg/mL)	1689.71 ± 855.91	1613.94 ± 682.53	0.55
Phosphate(mmol/L)	2.36 ± 0.53	2.11 ± 0.50	0.01
Cinacalcet(yes)	33.3%	46.3%	0.25
Intra-operative data			
Operation methods(PTX/RFA)	48/5	43/58	<0.01
Removed nodule numbers	3.94 ± 0.50	3.74 ± 0.56	0.02
Nodule’s maximum diameter (mm)	18.84 ± 5.78	19.64 ± 5.60	0.44
Operative duration(min)	115.49 ± 56.46	63.14 ± 75.41	<0.01
Post-operative data			
Postoperative pH	7.32 ± 0.04	7.28 ± 0.72	0.66
iPTH_d0_	98.57 ± 215.81	184.96 ± 268.60	0.07
iPTH_d1_	116.39 ± 274.03	154.47 ± 361.34	0.52
iPTH_d2_	106.06 ± 323.72	108.70 ± 191.16	0.98
iPTH_d3_	106.09 ± 258.51	113.32 ± 210.80	0.94
Ca_d0_	2.32 ± 0.23	2.34 ± 0.29	0.77
Ca_d1_	2.17 ± 0.31	2.18 ± 0.30	0.88
Ca_d2_	2.22 ± 0.29	2.19 ± 0.31	0.57
Ca_d3_	2.20 ± 0.26	2.19 ± 0.35	0.99
P_d3_	1.29 ± 0.40	1.17 ± 0.43	0.13

PTX, Parathyroidectomy; RFA, Radiofrequency Ablation; TnI, Troponin I; BNP, B-type natriuretic peptide; iPTH, intact parathyroid hormone; β-CTx, Beta C-terminal cross-linked telopeptides of typeIcollagen; N-MID, N-terminal osteocalcin; tPINP, total type I collagen N-terminal propeptide.

### Incidence of hyperkalemia

**Figure 1:**
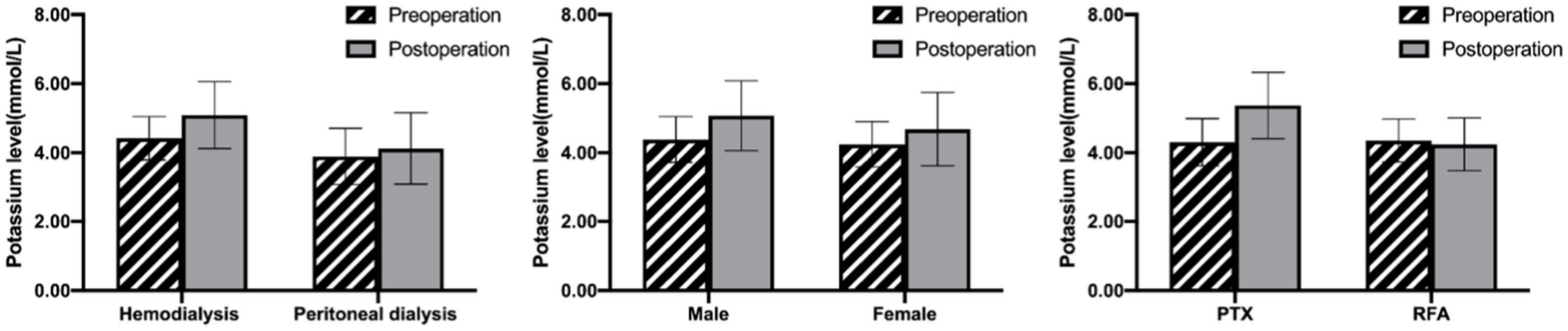
Comparison of the effect of dialysis mode **(A)**, sex **(B)** and operation methods **(C)** on serum potassium levels in ESRD patients undergoing invasive treatment. Data are presented as mean ± SD.

Serum potassium levels increased from the baseline of 4.32 ± 0.66 mmol/L to 4.90 ± 1.05 mmol/L after the operation. 79 patients (51.6%) were monitored serum potassium rose more than 0.5 mmol/L. There were 53 cases of postoperative hyperkalemia (serum potassium >5.3 mmol/L), and the incidence rate was 34.4%. The mean serum potassium level in the hyperkalemia group was 6.07 ± 0.49 mmol/L, which was higher than that in the non-hyperkalemia group (4.30 ± 0.67 mmol/L). There was no statistical difference between the two groups in several variables such as age, dialytic vintage, creatinine, UA, ALB, TNI, BNP, iPTH, Ca, Mg, pH, ALP (*p* > 0.05; Table 1). The preoperative serum potassium in the hyperkalemia group was 4.54 ± 0.74 mmol/L, which was significantly higher than that in the non-hyperkalemia group (4.21 ± 0.59 mmol/L; *p* < 0.01). There was also an obvious correlation with dialysis modality. The incidence of postoperative hyperkalemia in hemodialysis patients was significantly higher than that in peritoneal dialysis patients (38.4% vs. 17.2%, *p* = 0.03). Furthermore, it seemed that males were more prone to suffer hyperkalemia than females, but we found no statistically significant difference (40.9% vs. 25.8%, *p* = 0.05; Figures 1A,B). Although hyperkalemia did not cause fatal arrhythmia and other malignant events in our study, emergency hemodialysis was initiated, which increased additional expenses and injuries.

### Risk factors

According to the univariate logistic regression analysis of these variables, undergoing PTX, more parathyroid glands removed, longer duration of operation, and higher baseline serum phosphorus, Hb and 25(OH)D levels were correlated with the elevated risk of hyperkalemia after operation (Table 2). Logistic backward stepwise regression analysis showed that preoperative potassium levels (odds ratio (OR) 3.269, 95% confidence interval (CI) [1.638–6.534], *p* = 0.001), and therapeutic method as PTX (OR 18.119, 95% CI [5.716–57.438], *p* < 0.01) were independent risk factors associated with postoperative hyperkalemia.

**Table 2:** Univariate logistic regression of risk factors associated with postoperative hyperkalemia.

Parameters	OR(95% CI)	*p* value
Age(years)	0.732(0.979–1.030)	0.732
Gender(male)	2.034(1.004–4.120)	0.049
Dialysis vintage(years)	1.047(0.955–1.148)	0.330
Mode of dialysis(HD)	2.992(1.070–8.371)	0.037
Albumin(g/L)	1.031(0.957–1.111)	0.418
Hemoglobin(g/L)	1.018(1.002–1.036)	0.032
25(OH)D(ng/ml)	1.031(1.001–1.061)	0.043
Removed nodule numbers	2.246(1.044–4.830)	0.038
Operation methods(PTX)	12.949(4.754–35.267)	<0.01
Operative duration(min)	1.010(1.005–1.015)	<0.01
Potassium (mmol/L)	2.198(1.283–3.766)	0.004
Phosphate (mmol/L)	2.214(1.164–4.211)	0.015

### Subgroup analysis according to the operation methods

The above results revealed that the incidence of hyperkalemia in patients treated with PTX was significantly higher than that in patients treated with RFA (52.7% vs. 7.9%, *p* < 0.01; Figure 1C). Therefore, comparison of the two groups according to the operation methods revealed that there was no statistically significant difference in terms of general information (including age, gender, dialytic vintage and dialysis method) and preoperative variables (such as ALB, Hb, K, Ca, P, Mg, ALP, iPTH, pH levels, etc.; Table 3). Patients in the PTX group had more removed parathyroid gland nodules, longer operative duration, and a more significant decrease in iPTH levels compared with those in the RFA group (*p* < 0.05). But the differences in postoperative calcium and phosphorus levels between the two groups were not found. The average serum potassium level after PTX rose prominently, with an increase of 1.05 ± 0.92 mmol/L, whereas it was basically stable after RFA, with a fluctuation of −0.11 ± 0.78 mmol/L.

**Table 3:** Comparison of variables between PTX and RFA groups.

Parameter	PTX Group (*n* = 91)	RFA Group (*n* = 63)	*p* value
Pre-operative data			
Age(years)	51.22 ± 12.87	53.38 ± 14.52	0.33
Female:male(n/n)	37/54	29/34	0.51
Dialysis vintage(years)	7.97 ± 3.65	7.43 ± 3.56	0.36
Dialysis method, hemodialysis	84.62%	76.19%	0.19
Albumin(g/L)	36.69 ± 4.53	35.47 ± 4.62	0.11
Creatinine(μmol/L)	881.09 ± 252.20	833.04 ± 221.13	0.19
Uric acid (μmol/L)	419.22 ± 109.67	408.00 ± 99.46	0.52
TnI(μg/L)	0.04 ± 0.05	0.04 ± 0.03	0.96
BNP(pg/ml)	431.39 ± 701.55	700.09 ± 1015.38	0.10
Hemoglobin (g/L)	107.34 ± 20.05	102.57 ± 21.92	0.03
ALP(U/L)	419.03 ± 388.63	329.60 ± 274.16	0.87
β-CTx(pg/ml)	5383.08 ± 1019.96	5441.64 ± 983.36	0.73
N-MID(ng/ml)	258.59 ± 304.85	234.66 ± 60.44	0.56
tPINP(ng/ml)	1082.10 ± 288.01	1081.61 ± 254.58	0.99
25(OH)D(ng/ml)	23.67 ± 11.09	21.45 ± 12.83	0.27
Magnesium(mmol/L)	1.04 ± 0.21	1.07 ± 0.28	0.75
pH	7.41 ± 0.05	7.41 ± 0.05	0.41
Potassium (mmol/L)	4.30 ± 0.69	4.35 ± 0.63	0.67
Calcium (mmol/L)	2.43 ± 0.21	2.43 ± 0.27	0.99
iPTH (pg/mL)	1716.63 ± 786.35	1529.35 ± 671.08	0.13
Phosphate(mmol/L)	2.24 ± 0.53	2.15 ± 0.57	0.32
Cinacalcet(yes)	37.5%	47.2%	0.37
Intra-operative data			
Removed nodule numbers	3.91 ± 0.49	3.67 ± 0.60	<0.01
Nodule’s maximum diameter (mm)	18.93 ± 6.05	20.00 ± 5.03	0.27
Operative duration(min)	133.89 ± 47.35	4.05 ± 1.86	<0.01
Post-operative data			
Postoperative pH	7.25 ± 0.74	7.37 ± 0.04	0.20
Postoperative K	5.37 ± 0.96	4.24 ± 0.77	<0.01
iPTH_d0_	82.23 ± 198.87	240.14 ± 285.08	<0.01
iPTH_d1_	90.11 ± 234.93	216.58 ± 431.12	0.03
iPTH_d2_	82.08 ± 255.36	152.95 ± 233.36	0.42
iPTH_d3_	82.42 ± 210.15	172.90 ± 265.81	0.40
Ca_d0_	2.32 ± 0.25	2.35 ± 0.29	0.51
Ca_d1_	2.19 ± 0.32	2.16 ± 0.29	0.61
Ca_d2_	2.22 ± 0.31	2.15 ± 0.28	0.20
Ca_d3_	2.23 ± 0.34	2.12 ± 0.26	0.06
P_d3_	1.20 ± 0.40	1.23 ± 0.46	0.76

### ROC curve analysis

**Figure 2:**
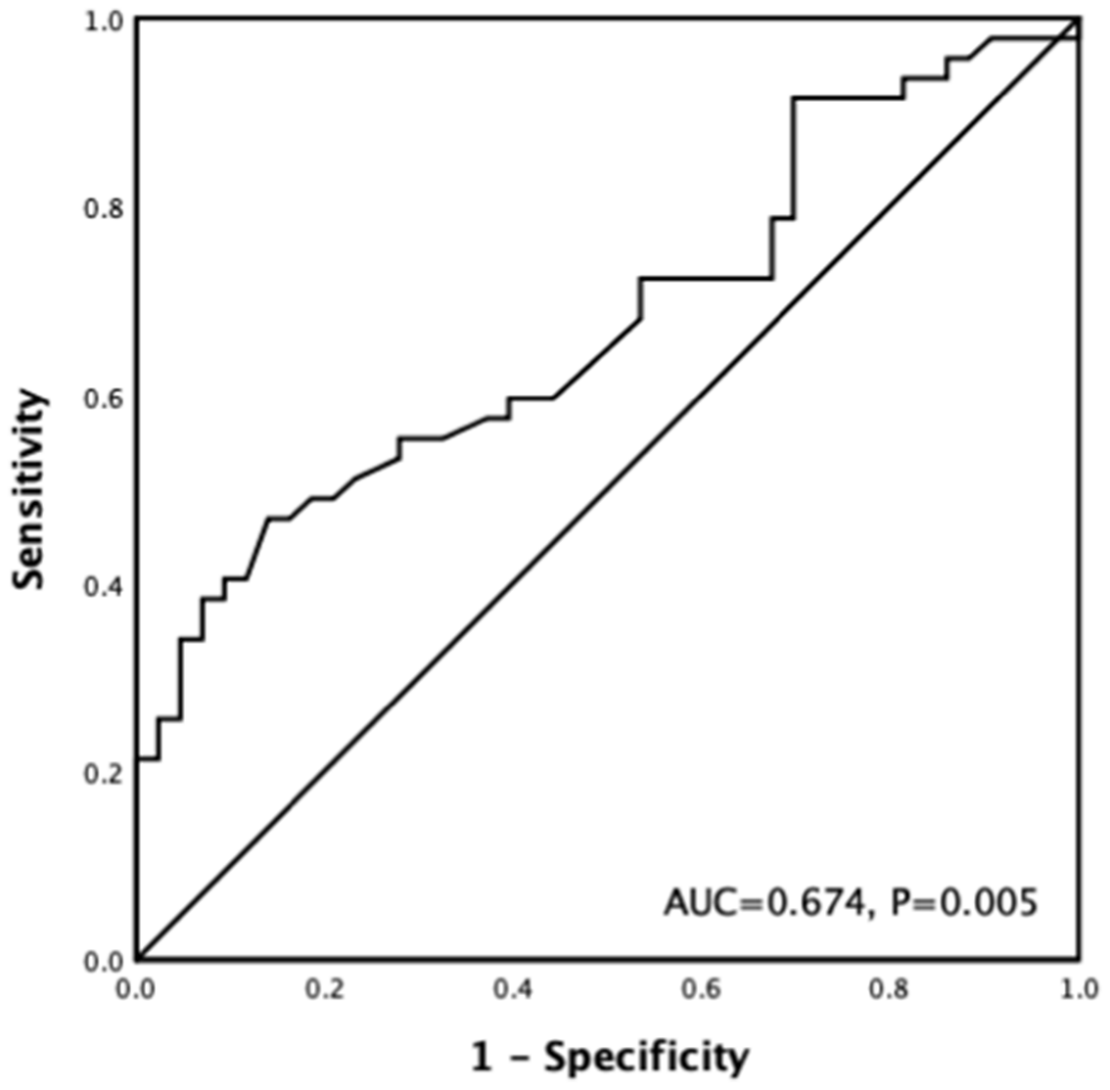
ROC curve of preoperative serum potassium associated with postoperative hyperkalemia after PTX.

According to ROC curve analysis, the optimal cutoff value of the preoperative serum potassium level for the prediction of the occurrence of hyperkalemia after the PTX operation was 4.66 mmol/L, and the area under the curve was 0.674 (*p* = 0.005), with a sensitivity of 46.8% and a specificity of 86% (Figure 2).

## Discussion

In our retrospective study, a total of 154 maintenance dialysis patients were treated with SHTP. And the incidence of postoperative hyperkalemia was found to be 34.4%, which was similar to previous studies reported (9–13). 79 patients (51.6%) had elevated serum potassium ≥0.5 mmol/L after the treatment, which was also close to a prospective study with a 58% incidence of increased potassium above 0.5 mmoL/L. The clinical manifestations of hyperkalemia are various, such as abnormal sensation in hands and feet, limb weakness, irritability, nausea or vomiting, and cardiac effect is the decrease of resting membrane potential (RMP) inducing either brady or tachyarrhythmic (16), even sudden death. SHPT lead to vascular calcification, myocardial fibrosis, and calcium deposition (17) due to the effects of persistent elevated PTH levels, volume overload, renal anemia, and uremic toxins, which results in decreased myocardial contractility and the multiplication of cardiovascular risks. It is more prone to sudden malignant arrhythmia due to sharply increased potassium levels (9). Postoperative hyperkalemia is relatively common, but its mechanism and risk factors have not been clarified, and further exploration is needed for perioperative potassium fluctuations and how to maintain potassium homeostasis.

The occurrence of postoperative hyperkalemia may be a multifactorial interaction. Combining the results of our study and previous reports, there are several potential influence factors.

Some studies (12, 18–21) indicated that higher preoperative serum potassium level was an independent predictor for postoperative hyperkalemia, which is consistent with the results of our study. And we found that the best preoperative potassium concentration to predict the occurrence of hyperkalemia after PTX was 4.66 mmol/L. If the potassium level before operation was monitored above this value, sodium zirconium cyclosilicate can be used preventively to increase the intestinal excretion of potassium, and intraoperative potassium levels should be monitored dynamically, so as to avoid or recognize hyperkalemia in time and reduce the incidence of adverse events.

The postoperative hyperkalemia was also significantly associated with the dialysis method. Hemodialysis patients had significantly higher serum potassium levels than peritoneal dialysis patients, both preoperatively and postoperatively. As peritoneal dialysis patients lose potassium from the peritoneal fluid, they usually have hypokalemia (22). In contrast, hemodialysis patients received 3 h heparin-free dialysis before treatment to prevent postoperative hemorrhage, which was shorter than the usual duration of 4 h, thus there was a risk of inadequate dialysis (23). Therefore, some studies suggested dialysis within 16–24 h preoperatively to reduce the risk of hyperkalemia (24).

Yang et al. (13) and Song et al. (18) found that the accident of hyperkalemia after operation increased in younger or male patients. And we also observed that the proportion of men was higher than that of women in the hyperkalemia group, compared with the non-hyperkalemia group, but the difference was not statistically significant (40.9% vs. 25.8%, *p* = 0.05). Younger or male patients have more muscles (9), and more muscle and peripheral tissue damage occurs during the operation, which exacerbates the risk of hyperkalemia by allowing a large efflux of potassium from intracellular compartment.

In addition, we found a significantly higher incidence of postoperative hyperkalemia in patients treated with PTX than in patients treated with RFA. PTX resulted in a more complete parathyroid nodules elimination due to more pronounced visualization exposure, accompanied by longer operative duration and greater tissue trauma. The mainstream explanation for the high prevalence of hyperkalemia after PTX is that, due to the more pronounced decline of iPTH levels after PTX than RFA, osteoclast activity decreases within a short period of time, and osteoblasts continue to function, resulting in rapid calcium intake by the skeleton and causing “hungry bone syndrome”(HBS) (14). The calcium ions in the extracellular fluid of the skeletal muscle cells drop sharply, and the Na^+^/Ca^2+^ Carrier (25) at the cell membrane promotes influx of sodium ions, which in turn activates the Na^+^/K^+^ ATPase pump (12, 26), leading to an increase in potassium ions in the extracellular fluid of skeletal muscle. Patients with uremia are unable to excrete and compensate the change of internal environment in time, which aggravates the development of life-threatening acute hyperkalemia. Several studies (11, 12, 20) indicated that predictors of HBS such as serum calcium and alkaline phosphatase levels (27) were independently associated with hyperkalemia after PTX. Unfortunately, we did not confirm its statistical correlation. The calcium supplementation immediately after operation and the frequency of perioperative monitoring of serum calcium cannot realistically reflect the actual dynamic changes of serum calcium *in vivo*.

Some drugs also induce hyperkalemia. Among the drugs used in general anesthesia surgery, such as the depolarizing neuromuscular blocker succinylcholine (28) or mannitol (29), as well as intravenous infusion of potassium-containing fluids, blood products or ACEIs/ARBs (30) during operation. Propofol infusion syndrome (31) has also been reported to trigger metabolic acidosis and severe hyperkalemia after heavy use of the general anesthetic propofol. Furthermore, it was noted that patients received prior treatment with cinacalcet (32) were more likely to develop postoperative hyperkalemia, speculating that it may be related to hypocalcemia.

More and more studies are now interested in ultrasound-guided radiofrequency ablation for the treatment of secondary hyperparathyroidism. RFA is uniquely superior to total parathyroidectomy in terms of minimally invasive, rapid, repeatable, safe and less complications (15, 33–35). Our previous study (15) noted that the differences between the two treatments were not statistically significant in outcomes, including all-cause mortality and cumulative response rate, and the incidence of postoperative hypocalcemia was similar. Several studies (4, 34, 36, 37) also concluded that RFA was no less effective than PTX, at least in the short-term follow-up time. Furthermore, our study firstly focuses on the difference in the occurrence of postoperative hyperkalemia between RFA and PTX.

There were several limitations in our study. As a single-center retrospective study, the number of patients is small. The changes of serum potassium, calcium and electrocardiogram were not monitored in real time during and immediately after operation, which led to delayed detection of perioperative hyperkalemia possibly; strict dietary potassium intake was not standardized, and the recirculation and adequacy of patients’ vascular access were not formally evaluated; limited treatment history of cinacalcet. Larger samples and prospective studies are needed for further exploration.

## Conclusion

In summary, patients with severe secondary hyperparathyroidism were susceptible to hyperkalemia after invasive treatment. The incidence of postoperative hyperkalemia after PXT was much higher than that of RFA, and hemodialysis patients were more prone to develop hyperkalemia, which was related to preoperative potassium levels. We suggest to achieve potassium levels below the threshold before surgery. The causes and mechanisms of postoperative hyperkalemia and its correlation with HBS need further study.

## Data Availability

The original contributions presented in the study are included in the article/Supplementary material, further inquiries can be directed to the corresponding author.
